# Effectiveness of an in-office intervention to improve general practitioners’ support for tobacco smoking cessation: results from a randomised controlled trial (TABAC-PRO)

**DOI:** 10.1186/s12875-025-03168-3

**Published:** 2026-01-09

**Authors:** Romain Guignard, Daisy Luangphinith, Alice Escande, Anysia Nguyen, Justine Avenel, Olivier Smadja, Anne Pasquereau, Viêt Nguyen-Thanh

**Affiliations:** 1 Santé publique France, the French national public health agency, Saint-Maurice, France; 2 The Behavioural Insights Team, Paris, France

**Keywords:** Smoking cessation, Health professional, General practitioners, Brief advice, Randomised controlled trial, Evaluation, Behavioural insights

## Abstract

**Background:**

Although support for tobacco smoking cessation provided by general practitioners (GP) has proven effective, such an approach remains quite rare. We designed a behavioural science-based intervention to improve GP support for patients to quit smoking. The present study aimed to evaluate the effectiveness of this in-office paper-based intervention by comparing it with a no-intervention control group.

**Methods:**

We conducted an online randomised controlled trial between January and May 2024 among GPs in France. The intervention consisted in sending a kit containing an information sheet for GPs outlining the main stages of brief advice for smoking cessation, questionnaires for patients asking them about their smoking status and, for those who smoked, their motivation to quit, as well as a poster to be displayed in each GP’s waiting room to encourage people who smoke tobacco to complete the questionnaire. The primary outcome was the proportion of people who smoke with whom GPs had discussed smoking cessation on their last full working day five weeks after the kit had been sent out. The secondary outcome was the likelihood that GPs provided cessation support (e.g., prescribed nicotine replacement therapy (NRT), proposed a follow-up consultation, and/or referred patients to other professionals) on the same day.

**Results:**

Of the 800 GPs recruited, 641 fully completed the final survey (333 in the intervention group and 308 in the control group). The proportion of people who smoke with whom GPs had discussed cessation was significantly higher in the intervention group (59.0% vs. 52.3%, *p* < 0.05). The likelihood of a follow-up consultation being offered to patients who smoke was also significantly higher in the intervention group (73.8% vs. 60.3%, *p* < 0.05). The likelihood of NRT prescription or referral were not significantly different between groups (82.4% vs. 77.9%, and 23.3% vs. 21.5%, respectively).

**Conclusions:**

Providing simple paper-based tools was associated with a greater likelihood of GPs discussing smoking cessation with their patients. This kind of intervention could usefully complement other primary care interventions, for example training, remuneration and financial incentives or care pathway reorganisation, with a view to significantly decreasing smoking prevalence.

**Trial registration:**

The study was retrospectively registered on 25 November 2024 (ISRCTN10207960).

**Supplementary Information:**

The online version contains supplementary material available at 10.1186/s12875-025-03168-3.

## Background

Tobacco is responsible for more than 8 million deaths worldwide every year [1, 2]. In 2020, 22.3% of the world’s population used tobacco in some form [3]. France first tackled tobacco consumption with the Veil law on 9 July 1976, which restricted tobacco advertising. This was followed by the Évin law on 10 January 1991, which banned smoking in different kinds of public places. In 2004, France ratified the World Health Organization’s (WHO) Framework Convention on Tobacco Control (FCTC), and since then, the country has implemented various smoking reduction programs, cancer plans and legislative actions (plain packaging, increased tobacco taxes and prices, reimbursement of nicotine replacement therapies (NRTs), etc.). Between 2000 and 2023, the proportion of people who smoke tobacco every day among 18–75 year olds in France decreased from 30.0% to 23.1% [4]. Despite these figures, smoking prevalence is still high, and higher than in most other European countries [3]. Accordingly, greater efforts are needed to reduce the prevalence of tobacco use even further.

In 2008, to assist the implementation of effective interventions to reduce the demand for tobacco at the country level, the WHO introduced a comprehensive policy package called MPOWER [5], which comprises six evidence-based strategies. One of these, called “Offering help to quit”, is a key step in significantly reducing tobacco consumption [6]. Health professionals (HPs) play a vital role in this strategy. Accordingly, smoking cessation training for HPs [7] and for medical students [8] is needed to improve their skills in smoking cessation management, and to encourage them to actively participate in providing cessation support. Moreover, other interventions have been designed to help HPs fully or partly implement the five A’s framework for smoking cessation (Ask, Advise, Assess, Assist, Arrange) [9] using text messages [10], training and other tools [11–13]. With a view to offering help to those who want to quit smoking, the French National Authority for Health (*Haute Autorité de santé*, HAS) strongly recommends that screening for tobacco consumption (i.e., Ask) be systematic for all patients, and that advice to quit smoking (i.e., Advise) be given for all patients who smoke. Previous studies highlighted that brief advice on quitting smoking could increase cessation success rates by 66% compared to no advice [14–16]. Even very brief advice (VBA), a type of intervention that essentially involves giving opportunistic advice to all patients who smoke, regardless of their motivation to quit smoking, in less than 3 min, for example using the AAR (Ask, Advise and Refer) method [17], has proven effective in increasing success rates by 17% according to a recent meta-analysis [18].

General practitioners (GPs) are the primary HPs in the French healthcare system and coordinate patients’ care pathways. Moreover, smoking cessation services are not as structured in France as in other countries, such as the United Kingdom [19]. Smoking cessation consultations are primarily provided by tobacco specialists (health professionals with a diploma in tobacco addiction) in hospital settings, meaning they are not necessarily located near homes of people who smoke. This is why smoking cessation support also relies heavily on GPs. Nevertheless, according to a representative French national survey carried out in 2021, among people who smoke who had consulted at least one HP in the previous twelve months, only 22.5% had discussed tobacco use with an HP, and only 8% of these at an HP’s initiative [20]. These results contrast with those of a previous survey conducted among GPs in 2019–2020, in which 66% of the participants reported they systematically screened for tobacco smoking and performed repeat screening for each patient [21]. Part of the difference could be due to the fact that screening smoking status in general practice is not systematically followed by a discussion about smoking habits or quitting. All these results highlight the need to improve the role of GPs in France in helping patients quit smoking and to support them in their practice, especially given that nearly 60% of people who smoke every day in the country want to quit, that between 25 and 30% try to quit smoking at least one week each year [22], and that over 60% of people who try to quit do so without any help [23].

The literature highlights various behavioural hypotheses from HPs’ (especially GPs) and patients’ perspectives about potential barriers to smoking cessation in patients and to the provision of related support. For HPs, these barriers include (i) time constraints, (ii) a lack of training, (iii) the belief that any support they provide is ineffective, (iv) the opinion that patients are not motivated to quit, and (v) their fear of upsetting patients if they bring up the subject [24–27]. For patients, in particular those with a low socioeconomic status (the subgroup with the highest smoking prevalence, especially in high income countries [28–30]), barriers include (i) the fact that other needs and objectives are prioritized over smoking cessation, (ii) low self-confidence about being able to quit, (iii) the fact that tobacco smoking is still considered a social norm in some groups, (iv) a lack of social support, (v) the belief that quitting depends only on individual will, and (vi) negative attitudes about the support provided by HPs [31, 32].

The present study evaluated the effectiveness of an intervention addressing specific behavioural barriers (for GPs: low perceived self-efficacy in supporting cessation, underestimation of patients’ motivation to quit, fear of upsetting them; for patients who smoke: negative attitudes about support, low perceived self-efficacy in quitting; for both: the tendency to wait for the right moment) to increase discussion on smoking cessation between patients and their GPs, compared with no intervention.

## Methods

### Trial design

This single-blind randomised controlled trial study was conducted between January 2024 and May 2024. The intervention group received a kit comprising several paper-based tools while the no-intervention control group received no kit during the study period. The present article follows the CONSORT 2010 guidelines. The protocol study was registered on the ISRCTN registry (n° ISRCTN10207960).

### Participants and procedure

We included GPs working in France in office-based practice (i.e., GPs who exclusively worked in facilities where one can spend at least one night like private or public hospitals or social care facilities, in private companies, or in schools were excluded). We recruited participants via a specialised panel provider (B3TSI) and from a file of additional GPs who had previously agreed to participate in future research studies. Recruitment took place between January and March 2024. Participants filled in an online recruitment questionnaire which collected baseline data (including sociodemographic characteristics, details about their current general practice, and screening and management practices for different risky behaviours) (supplementary file, Appendix 1). Two months after recruitment, a final questionnaire was sent via email to all participants (supplementary file, Appendix 2). It was developed by the research team for this study.

GPs who completed the final questionnaire were given a financial compensation of 30 euros. To maximise participation, up to eight reminders were sent to those who did not fill in the questionnaire.

### Participants and procedure: modification to protocol

The number of GPs recruited between January and March 2024 fell short of the calculated minimum required sample size. Accordingly, we modified the original protocol to include three other recruitment channels. First, banners providing information about the study were placed on more than 600 commonly used internet sites (e.g., meteofrance.com, youtube.com). Second, on its website, the National Health Insurance displayed a link redirecting internet users to the study. It also promoted the study via its newsletter for all French doctors. Finally, the College of General Medicine (CMG) relayed the study to its member organizations via another web link.

### Intervention

The intervention presented below was first tested for feasibility and GP and people who smoke acceptability before being implemented [33].

The intervention comprised a kit of three paper-based documents: an information sheet for GPs on how to carry out a very brief smoking cessation advice activity in three stages (Ask, Advise and Refer), two hundred copies of a two-sided page with the patient questionnaire to assess tobacco consumption and attitudes towards quitting on the front, and an information note on quitting on the back, and a poster designed for waiting room encouraging patients to fill in the questionnaire (supplementary file, Appendix 3). These tools were designed to act on several behavioural levers:

- GP information sheet: This document was designed to provide a simple heuristic for GPs, outlining three key steps to approach smoking cessation discussions. Specifically, it served as a visual reminder to initiate conversations about smoking cessation, to reassure doctors who felt uncertain about addressing the topic, and to reinforce the importance of their role in supporting patients to quit.
- Patient questionnaire: The questionnaire was designed to facilitate spontaneous discussion about smoking. It included questions about tobacco use, heaviness of smoking (thanks to two questions on self-reported time to the first cigarette of the day and number of cigarettes smoked per day) [34], previous quit attempts (number and use of nicotine replacement therapy) and willingness to quit. Its aim was twofold: to prompt patients to reflect on smoking cessation just before their appointment with the GP, and to indicate to GPs which patients were interested in quitting. The latter element was particularly important, as GPs often underestimate patients’ motivation to quit and may avoid the topic for fear of upsetting them. An information section on the back of the questionnaire sheet explained both the short- and long-term benefits of quitting, as well as the role of GPs in and their potential impact on smoking cessation. The aim of providing this information was to reinforce patient’s perceived self-efficacy in smoking cessation.
- Waiting room poster: The aim of this poster was to encourage patients to complete the questionnaire and to hand it to their GPs during their consultation.

The questionnaire and poster also aimed to signal clearly to patients that their GPs were available to discuss smoking cessation.

An implementation guide was included within the kit, explaining how to display and use the three different elements. This guide recommended leaving the questionnaires in the waiting room or having them distributed by reception staff, and then asking the patient, after their consultation, if they had seen or filled in the questionnaire. Irrespective of their answer, this question helped initiate a discussion on smoking cessation using the information sheet.

The kit was sent by post to GPs allocated to the intervention group; they were asked to test the tools for four weeks.

### Outcomes

Outcomes were collected in the final questionnaire (which was sent about five weeks after the kits were posted) (supplementary file, Appendix 2). The primary outcome was the number of people who smoke with whom the GP discussed smoking cessation on his/her last full working day. The secondary outcome was the likelihood that they provided cessation support to people who smoke on this same day. There were also several exploratory outcomes: (i) for GPs who provided cessation support, the type of support provided from the following options: NRT prescription, referral to another HP, a subsequent follow-up consultation dedicated to smoking cessation; (ii) in terms of GPs’ cessation support activities during the previous two working weeks, the proportion of people who smoke (a) whose level of tobacco dependence they assessed, (b) whose motivation to quit smoking they assessed, (c) who they advised to reduce tobacco consumption if they did not want to quit, and (d) who they reminded about the benefits of quitting smoking. For the latter four (i.e., a-d) outcomes, the answer options were: 0% (i.e., no patient), less than 10%, 10 to 24%, 25 to 49%, 50 to 74%, 75 to 90%, and all patients.

### Outcomes: modification to protocol

The primary outcome was modified to the proportion (i.e., not the number) of patients who smoked with whom the GP discussed smoking cessation on his/her last full working day. This modification was made to take into account the fact that some GPs had not seen any people who smoke on their last full working day (*n* = 10). This change did not impact the study’s statistical power.

### Sample size calculation

To detect a minimum of one extra cessation discussion a day in the intervention group at 0.05 significance level, with a power of 80%, a standard deviation of 5 and an assumed attrition of 50%, we calculated that 1500 GPs needed to be recruited.

### Sample size calculation: modification to protocol

We performed a new statistical power calculation which revealed that a sample of 750 GPs answering the final questionnaire could detect a 10.16 percentage point increase in the proportion of patients with whom a doctor discussed smoking cessation on their last full working day (i.e., modified primary outcome).

### Randomisation

After they completed the online recruitment questionnaire, GPs were randomised into the intervention and control groups using an integrated algorithm. Participants were not made aware of their treatment allocation. While GPs in the intervention group were immediately sent the kits, GPs in the control group were sent an email with the following carefully worded sentences: “We shall contact you again you by e-mail in March to invite you to fill in a questionnaire about your current practices. After this, we shall send you the tools we have created.” This wording was chosen to ensure, as much as possible, that GPs in this group were unaware of the group they had been randomised into.

### Statistical methods

We used linear regression to analyse the (modified) primary outcome. The control variables were age (categorical), gender (categorical), geographical area (categorical), practice type (e.g., individual, group, health centre) (categorical), number of patients consulted during the last full working day (continuous), employment status (part-time/full-time) (binary), smoking status (categorical), baseline smoking-related medical practices (categorical), day of survey completion (categorical), presence of another doctor in the practice who also participated in the study (yes/no) (binary).

The secondary and exploratory analyses used the same specifications as the primary analysis, but with a logistic regression.

We hypothesized that despite including an implementation guide in the kit, not all the participants in the intervention group would use it as intended. Accordingly, we performed a compliance analysis. Specifically, we defined compliance as receiving the kit, reading the information sheet and using the patient questionnaire (i.e., distributed in the waiting room, used in conversation, handed out directly by the GP or medical secretary). We calculated the Local Average Treatment Effect (LATE) by dividing the average effect of the intervention on intention to treat by the proportion of participants who were compliant.

### Statistical methods: modification to protocol

For each of the four exploratory outcomes concerning their previous two weeks of work, we created a binary variable illustrating whether or not they had provided support to at least half of their patients who smoked.

## Results

### Baseline data

**Fig. 1:**
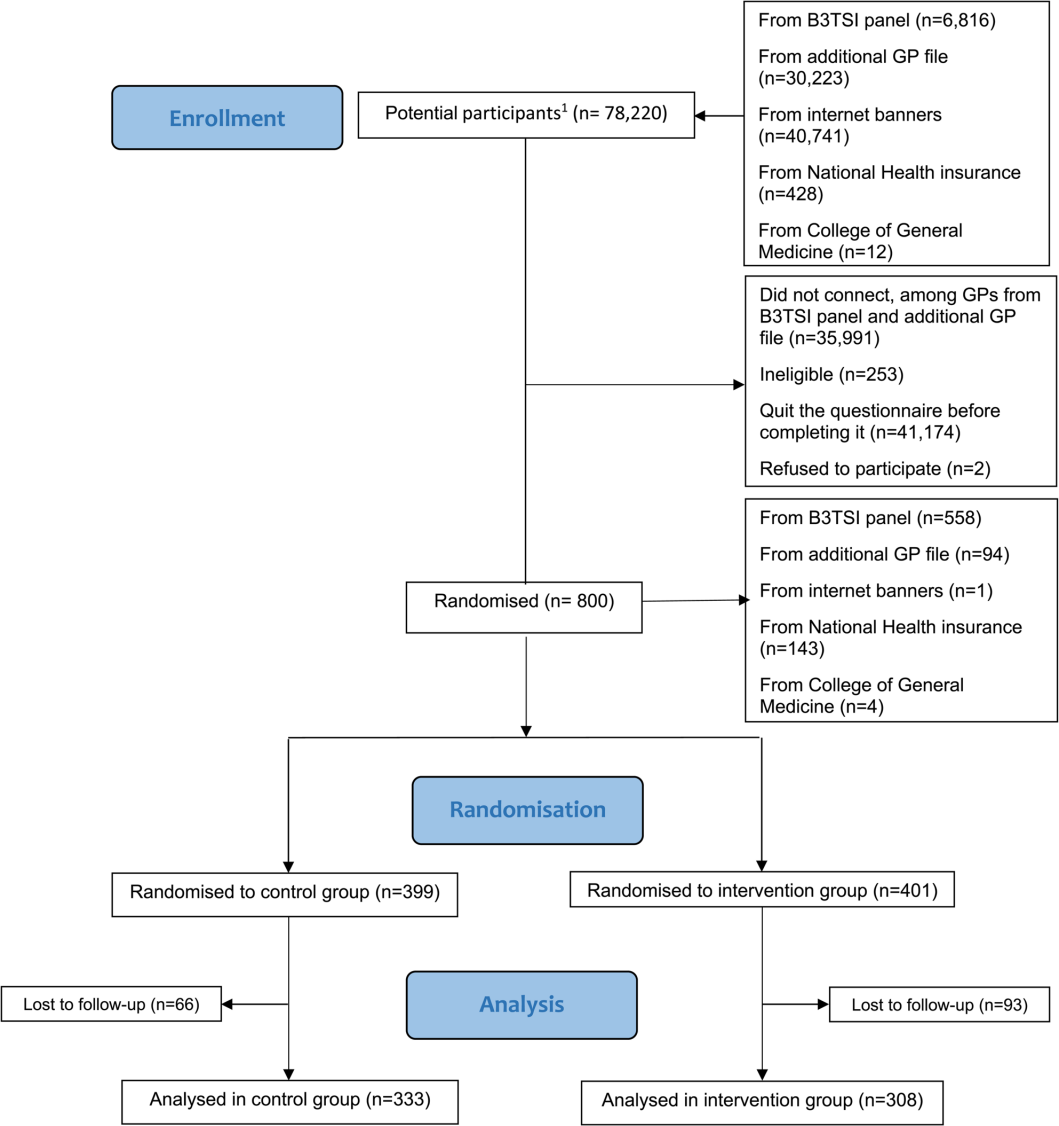
Flow diagram for the Tabac-Pro randomised controlled trial

We sent an invitational email to 37,039 GPs through the B3TSI panel and the file of additional GPs who had previously indicated their interest in participating in research studies. With regard to the extra three recruitment channels set up at a later stage, 40,741 persons clicked on the internet banners and 440 persons clicked on the web links distributed by the National Health Insurance and the College of General Medicine. There were therefore a total of 78,220 potential participants, some of whom may have been contacted several times because of the various channels used. Of these, 35,991 did not open the recruitment questionnaire (all from the B3TSI panel and the additional GP file) and 41,174 quit the questionnaire before completing it. Moreover, 253 respondents were ineligible and two refused to participate. The remaining 800 GPs who agreed to participate and who completed the recruitment questionnaire constituted the recruited sample (70% of them came from B3TSI panel). Among them, 401 GPs were randomised to the intervention group and 399 GPs to the control group. Of these, 308 and 333, respectively, completed the final questionnaire, making a final study sample of 641 GPs. For the analysis concerning people who smoke consulted by GPs on their last full working day, 10 GPs were excluded because they did not see any such patient that day (three in the control group and seven in the intervention group) (Fig. 1).

### Attrition

GPs who did not complete the final questionnaire were older (52.1 years old vs. 49.0 years old for those who did complete it, *p* < 0.05), were more likely to work in a public or private hospital, or in a nursing home (6.9% vs. 3.1%, *p* < 0.05), were slightly more likely to screen for alcohol consumption (33.3% did so systematically and repeatedly for each patient vs. 29.5%, *p* < 0.05), and were more likely to systematically talk about smoking cessation when they learned their patients smoked (52.2% vs. 40.9%, *p* < 0.05). There was no difference between the two groups concerning the frequency of screening for smoking status.

### Sample description

Among the 641 participants, 43.8% were women, mean age was 49.0 years, and 93.5% did not smoke tobacco at the time of the study. Half (52.6%) were working in urban areas with more than 100.000 residents and 18.1% in rural areas. Only 32.3% had an individual practice. GPs worked an average of 40.9 h and an average of 4.3 days a week. More than half (56.2%) declared that they systematically and repeatedly screened for their patients’ smoking status, while 40.9% declared that they talked systematically about smoking cessation with patients who smoke (Table 1). There was no significant difference between the intervention and control groups concerning variables collected at baseline.

**Table 1:** Sample description according to the randomisation group

	Control	Intervention	Total
	*N* = 333	*N* = 308	*N* = 641
	N (%)	N (%)	N (%)
**Gender**			
Man	194 (58.3%)	164 (53.3%)	358 (55.9%)
Woman	138 (41.4%)	143 (46.4%)	281 (43.8%)
Other	N/A	N/A	N/A
**Mean age (SD)**	49.1 (11.9)	49.0 (11.1)	49.0 (11.5)
**Do you smoke?**			
Yes, I smoke every day	10 (3.0%)	8 (2.6%)	18 (2.8%)
Yes, I smoke occasionally	11 (3.3%)	13 (4.2%)	24 (3.7%)
No, I do not smoke, but I have smoked in the past	105 (31.5%)	86 (27.9%)	191 (29.8%)
No, I have never smoked	207 (62.2%)	201 (65.3%)	408 (63.7%)
**Type of agglomeration**			
Rural	61 (18.3%)	55 (17.9%)	116 (18.1%)
Fewer than 20,000 inhabitants	60 (18.0%)	51 (16.6%)	111 (17.3%)
20,000 to 99,999 inhabitants	38 (11.4%)	39 (12.7%)	77 (12.0%)
100,000 inhabitants or more	123 (36.9%)	123 (39.9%)	246 (38.4%)
Paris agglomeration	51 (15.3%)	40 (13.0%)	91 (14.2%)
You are currently working *…			
In an individual practice	114 (34.2%)	93 (30.2%)	207 (32.3%)
In a group practice with other doctors (GPs or specialists)	117 (35.1%)	126 (40.9%)	243 (37.9%)
In a multi-professional group practice (e.g., doctors, nurses, physiotherapists, midwives) with no common health mission	48 (14.4%)	43 (14.0%)	91 (14.2%)
In a multi-professional medical clinic with a common health mission (called a *maison de santé pluri-professionnelle*) or a health centre	70 (21.0%)	57 (18.5%)	127 (19.8%)
In another type of healthcare establishment (hospital, clinic, nursing home, etc.)	11 (3.3%)	N/A	20 (3.1%)
In a company, school or association	N/A	N/A	N/A
**You are currently working …**			
Only in a private practice	277 (83.2%)	246 (79.9%)	523 (81.6%)
Only as an employee	20 (6.0%)	19 (6.2%)	39 (6.1%)
In private practice and as an employee (mixed activity)	36 (10.8%)	43 (14.0%)	79 (12.3%)
Mean number of patients consulted per day (SD)	28.0 (8.5)	28.1 (9.4)	28.0 (8.9)
Mean number of consultation hours worked per week (SD)	41.2 (12.2)	40.6 (13.5)	40.9 (12.8)
Mean number of consultation days per week (SD)	4.4 (0.9)	4.3 (0.9)	4.3 (0.9)
**Do you ask about alcohol consumption…?**			
Systematically and repeatedly for each of your patients	95 (28.5%)	94 (30.5%)	189 (29.5%)
Systematically and only once per patient	105 (31.5%)	81 (26.3%)	186 (29.0%)
Only for certain patients you consider to be at risk	132 (39.6%)	133 (43.2%)	265 (41.3%)
You never ask about it	N/A	N/A	N/A
**Do you ask about physical activity…?**			
Systematically and repeatedly for each of your patients	155 (46.6%)	139 (45.1%)	294 (45.9%)
Systematically and only once per patient	81 (24.3%)	67 (21.8%)	148 (23.1%)
Only for certain patients you consider to be at risk	96 (28.8%)	102 (33.1%)	198 (30.9%)
You never ask about it	N/A	N/A	N/A
**Do you ask about smoking status…?**			
Systematically and repeatedly for each of your patients	184 (55.3%)	176 (57.1%)	360 (56.2%)
Systematically and only once per patient	108 (32.4%)	87 (28.3%)	195 (30.4%)
Only for certain patients you consider to be at risk	41 (12.3%)	45 (14.6%)	86 (13.4%)
You never ask about it	0	0	0
**If you learn that a patient smokes**,** do you talk to him/her about quitting?**			
Never	0	0	0
Rarely	N/A	N/A	N/A
Sometimes	16 (4.8%)	18 (5.8%)	34 (5.3%)
Often	88 (26.4%)	78 (25.3%)	166 (25.9%)
Very often	93 (27.9%)	84 (27.3%)	177 (27.6%)
Always	136 (40.8%)	126 (40.9%)	262 (40.9%)

In accordance with the UK Office for National Statistics guidelines and to protect the anonymity of respondents, any category containing fewer than 10 people is not reported

*The ‘type of practice’ characteristic was a multiple-choice question; this is why the sum of the numbers provided does not correspond to 641

### Outcomes

On their last full working day, GPs consulted a mean of 26.5 adult patients; a mean of 6.9 were people who smoked tobacco and, among them, 3.8 discussed smoking cessation with their GPs (Table 2).

**Table 2:** Average number of patients who smoke who received different kinds of support from general practitioners on their last full working day, according to the randomisation group

		Control	Intervention	Total
		*N* = 333	*N* = 308	*N* = 641
Number of patients (SD)		26.6 (8.3)	26.3 (9.4)	26.5 (8.8)
Number of patients who smoke (SD)		7.0 (4.5)	6.9 (4.3)	6.9 (4.4)
Number of patients who smoke with whom GPs discussed smoking cessation (SD)		3.6 (3.2)	4.0 (3.2)	3.8 (3.2)
Number of patients who smoke who received NRT prescription* (SD)		2.8 (2.8)	2.8 (2.7)	2.8 (2.7)
Number of patients who smoke who were proposed a follow-up consultation* (SD)		2.2 (2.7)	2.5 (2.7)	2.4 (2.7)
Number of patients who smoke who were referred to other health professional* (SD)		0.5 (1.4)	0.5 (1.4)	0.5 (1.4)

SD: standard deviation; GP: general practitioner; NRT: nicotine replacement therapy

*Question asked to general practitioners who discussed smoking cessation with at least one patient (*n* = 576)

The proportion of people who smoke with whom GPs had discussed smoking cessation was significantly higher in the intervention group compared with the control group (Beta = 6.73; predicted value: 59.0% vs. 52.3%; *p* < 0.05), corresponding to a 13% relative increase between the two groups (Table 3).

**Table 3:** Linear regression for the proportion of people who smoke with whom GPs discussed smoking cessation on the last full working day (*N* = 631)

	Beta estimates (standard error)	*P*-value
**Intervention group**	6.73 (2.66)	0.012
**Age** (ref. = 28–40)		
41–50	1.48 (3.67)	0.687
51–60	3.71 (3.79)	0.328
61 +	1.53 (4.42)	0.730
**Gender** (ref. = Man)		
Woman	2.15 (2.81)	0.444
Other	-2.57 (24.18)	0.915
**Geographical area of residence** (ref. = rural area)		
Urban area	-3.45 (3.07)	0.261
Periurban area	-0.68 (4.20)	0.872
**Practice type**		
Individual practice	0.82 (6.67)	0.902
Group practice with other doctors	-3.46 (6.33)	0.585
Multi-professional group practice with no common health mission	-2.08 (6.20)	0.738
Multi-professional medical clinic with a common health mission (*Maison de santé pluri-professionnelle)* or a health centre	-7.16 (6.57)	0.276
Other healthcare establishment	-2.50 (8.55)	0.770
Company / School / Association	-4.37 (13.02)	0.737
Number of patients consulted on last full working day	0.07 (0.16)	0.681
Full-time job (ref. = part-time job)	-3.72 (3.43)	0.278
**Smoking status** (ref. = Never smoked)		
Currently smoke	-3.32 (5.01)	0.508
Used to smoke	1.03 (3.10)	0.739
**Screening for patients’ smoking status** (ref. = Systematic and repeated)		
Systematic but only once	-13.54 (3.07)	0.000
Only for perceived at-risk patients	-15.09 (4.33)	0.001
Frequency of discussions about smoking cessation	18.19 (5.02)	0.000
**Day of survey completion** (ref. = Sunday)		
Monday	6.70 (9.57)	0.484
Tuesday	0.74 (9.78)	0.939
Wednesday	7.86 (9.64)	0.415
Thursday	5.91 (9.62)	0.539
Friday	7.10 (9.61)	0.460
Saturday	12.85 (10.67)	0.229
Presence of another doctor in the practice who also participated in the study	1.42 (5.34)	0.791
Intercept	37.15 (15.13)	0.014

Estimates were computed with all variables included in the linear regression

Overall, the proportion of GPs offering some form of smoking cessation support to patients who smoke was not significantly different between both groups (89.4% in the intervention group vs. 83.3% in the control group, *p* > 0.05). In terms of each type of support, the likelihood of NRT prescription and referral to another HP were not significantly different between both groups (respectively 82.4% vs. 77.9% and 23.3% vs. 21.5%, *p* > 0.05). However, a significantly higher proportion of GPs from the intervention group offered a follow-up consultation to discuss smoking cessation (73.8% vs. 60.3%, *p* < 0.05).

With regard to exploratory variables concerning cessation-related practices over the previous two weeks, the proportion of GPs who evaluated tobacco dependence for at least half of their patients who smoked was significantly higher in the intervention group (33.4% vs. 24.3%, *p* < 0.05). Moreover, the proportion of GPs who assessed patients’ motivation to quit was notably, albeit not significantly, higher in the intervention group (51.3% vs. 43.2%, *p* = 0.056). There was no significant difference between the two groups concerning advice to reduce consumption and reminding patients of the benefits of smoking cessation (Table 4).

**Table 4:** Results for secondary and exploratory outcomes: observed percentages and p-values for intervention effect obtained from multiple logistic regressions (*N* = 641)

	Control group (*n* = 333)	Intervention group (*n* = 308)	*P*-value for difference
Overall proportion of GPs who provided smoking cessation support on last full working day	83.3%	89.4%	0.087
Proportion of GPs who prescribed NRT on last full working day	77.9%	82.4%	0.299
Proportion of GPs who proposed a follow-up consultation on cessation on last full working day	60.3%	73.8%	0.001
Proportion of GPs who referred patients to other health professionals on last full working day	21.5%	23.3%	0.637
Proportion of GPs who, in the previous two weeks, assessed level of tobacco dependence in at least half of their patients who smoked	24.3%	33.4%	0.012
Proportion of GPs who, in the previous two weeks, assessed motivation to quit in at least half of their patients who smoked	43.2%	51.3%	0.056
Proportion of GPs who, in the previous two weeks, advised at least half of their patients who smoked and did not want to quit, to reduce their tobacco consumption	56.5%	62.7%	0.110
Proportion of GPs who, in the previous two weeks, reminded at least half of their patients who smoked of the benefits of quitting	61.9%	65.3%	0.543

*P*-values were computed using logistic regressions adjusted for age (categorical), gender (categorical), geographical area of residence (categorical), practice type (categorical), number of patients consulted during the last full working day (continuous), employment status (part-time/full-time) (binary), smoking status (categorical), baseline smoking-related medical practices (categorical), day of survey completion (categorical), presence of another doctor in the practice who participated in the study (binary). GP: general practitioner; NRT: nicotine replacement therapy

Among GPs randomised into the intervention group, 12% reported they did not receive the intervention kit. The vast majority of those who received the kit reported they read the information sheet (97.9%) and used the patient questionnaire (91.9%). A majority also displayed the poster (65.7%). The compliance analysis highlighted that when used, the intervention tools increased the proportion of patients with whom physicians discussed smoking cessation by 8.6% points (from 52.3% to 60.9%).

## Discussion

The provision of simple paper-based tools to support smoking cessation was effective in encouraging GPs discuss this topic more with their patients. If we express our results in absolute terms, the higher percentage of smoking cessation discussions in the intervention group corresponds to 2.5 extra discussions per week per GP. The impact of the intervention may seem weak, but its implementation is quite simple (hanging the poster, read the information sheet, making the questionnaires available) and not time-consuming for GPs before initiating a discussion on smoking cessation. It also relies on patients who must take the initiative to fill out the questionnaire. The qualitative pre-test we conducted before the present study demonstrated its acceptability among GPs [33]. Finally, even with a relatively low impact at an individual level, if this intervention were disseminated to a greater number of GPs, these extra discussions could substantially impact smoking behaviours in France, given that previous studies found that brief advice from a physician increases the chances of quitting by 66% [15]. Another result from this study is that our intervention was associated with an increased likelihood of a follow-up consultation focused on cessation.

Overall, this intervention aimed to bridge the gap between GPs, who tend to underestimate the proportion of their patients interested in quitting smoking, and patients, who lack adequate professional support to help them. Our findings suggest that enabling patients to clearly signal their desire for support, and equipping GPs with simple, evidence-based tools for brief interventions, can significantly increase the number of discussions about smoking cessation, ultimately fostering more opportunities for effective support and behavioural change. It should be noted that the mere receipt of this material by GPs in the intervention group may have reinforced their perceived role in supporting their patients to quit smoking. This intervention seeks to complement others such as training, remuneration and financial incentives [35], and care pathway reorganisation [36]. We also sought to make this intervention user-friendly in terms of its implementation. We hypothesize that the information sheet and patient questionnaire enabled GPs to identify a greater number of patients interested in talking about smoking cessation and to structure related discussions more effectively. Additionally, we hypothesize that these tools facilitated more in-depth conversations, allowing GPs to comprehensively assess patients’ level of dependence, and to explore appropriate support options, including follow-up consultations.

The fact that the only support more often provided in the intervention group was a follow-up consultation, and not the prescription of nicotine replacement therapy (NRT) nor referral to another healthcare professional, is not easy to interpret, given that these three actions were mentioned in the information sheet. It is likely, however, that this reflects the intervention’s primary objective that was to initiate discussions about smoking cessation, and that a follow-up consultation emerged as a solution to the lack of time dedicated to this discussion during a consultation for another reason.

Our randomised control trial showed significant and positive results. Moreover, besides being able to target various behavioural barriers, the designed intervention is easily implementable. Several types of HPs can prescribe NRTs in France: doctors, midwives, occupational physicians, dental surgeons, nurses and physiotherapists. If this intervention were adapted and applied to a wider number of HPs, smoking cessation could be tackled simultaneously at different points along the care pathway. However, it is important to point out that many HPs who can legally prescribe NRTs are not trained in this practice. Accordingly, this intervention would most probably not be intensive enough for them and prior training would be necessary.

This study has several limitations. First, although our sample was quite similar to the general population of GPs in France in terms of age and gender, it differs in certain aspects, which could affect the generalizability of our results. Specifically, very few participants smoked tobacco (6.5%) and almost two-thirds had never smoked (63.7%). These figures contrast a representative study conducted in 2015, which found that 16% of French GPs smoked tobacco [37]. Another study showed that HPs who smoked were less likely to initiate smoking cessation interventions compared to their non-smoking counterparts [38]. Accordingly, our intervention may be less effective for GPs who smoke. Second, although we carefully worded the recruitment e-mail in order to ensure blinding, participants may have realised which group (i.e., intervention or control) they had been randomised to. Third, social desirability and memory biases cannot be excluded and may be different between both groups, since GPs from the intervention group may recall more smoking cessation advice by having received the material. However, main outcomes relate to the last full day worked, which minimizes the risk of bias. Fourth, our sample mostly came from the B3TSI panel, a platform for HPs to participate in surveys in exchange for financial compensation. Although we did not investigate any related characteristic, it is possible that the GPs included in the study were particularly interested in innovation and in seeking improvement in their practice. Among the strengths of the study, the intervention was designed based on behavioural sciences and a review of the barriers and levers to smoking cessation and its management among people who smoke and HPs. Its effectiveness was rigorously evaluated using a randomised controlled trial.

In conclusion, our intervention could complement other smoking cessation interventions (e.g. the French *Stoptober-like* campaign [39] or training of health professionals) by encouraging GPs to have more discussions about smoking cessation with their patients. This type of intervention contributes to the “Offering help to quit” axis of the MPOWER strategy promoted by the WHO, and combined with other MPOWER measures aimed at the environment and the general public, it has the potential to contribute reducing smoking prevalence in France.

## Supplementary Information

Supplementary Material 1.

## Data Availability

The datasets used and analysed in the current study are available from the corresponding author on reasonable request. Anonymized data can be shared with external teams after analysis by Santé publique France of submitted projects and after the signing of a commitment to confidentiality.
