# Supplementary material for: Effectiveness of an in-office intervention to improve general practitioners’ support for tobacco smoking cessation: results from a randomised controlled trial (TABAC-PRO)
Source: BMC Prim Care. 2026 Jan 9;27:49. doi: 10.1186/s12875-025-03168-3 (PMC12882597; doi:10.1186/s12875-025-03168-3)
Supplement: Supplementary file 1 — Supplementary Material 1. [file 12875_2025_3168_MOESM1_ESM.pdf]

Appendix 1: RECRUITMENT QUESTIONNAIRE

Take part in a study to encourage smoking cessation among your patients.

Thank you for your interest in this study, which aims to evaluate tools designed to help you better support your patients in quitting smoking. These easy-to-use tools have been developed to save you time.

To register, simply complete this questionnaire in full, which will take less than 3 minutes.

Please note that you cannot go back to change your answers. Be sure to complete the questionnaire to the end for your responses to be recorded.

QR1. Are you a general practitioner?

1. Yes
2. No -> END OF QUESTIONNAIRE

QR2. You are currently working in…

Multiple answers possible

1. An individual practice
2. A group practice with other doctors (GPs or specialists)
3. A multi-professional group practice (doctors, nurses, physiotherapists, midwives, etc.)
4. A multi-professional health centre (MSP, *maison de santé pluriprofessionnelle*), health centre, or health hub
5. Another type of healthcare establishment (hospital, clinic, nursing home, etc.)
6. A company, school, or association

QR3. Do you practice in metropolitan France?

1. Yes
2. No -> END OF QUESTIONNAIRE

If eligible

QR4. Do you agree to participate in this trial?

1. I have read the information and agree to participate
2. No -> END OF QUESTIONNAIRE

QR5. What is your RPPS number?

QR6. Please provide the following information about your main practice or workplace, which is essential for the study and for sending the tools:

These easy-to-use tools have been designed to save you time.

Last name: ____________

First name: ____________

Practice address (number, street): __________________

Apartment, building (optional): _______________

Postal code:

City: _________________

QR7. You are currently working…

1. Only in a private practice
2. Only as an employee
3. In a private practice and as an employee (mixed practice)

QR8. In which sector do you practice?

1. Sector 1
2. Sector 2
3. Non-contracted

QR9. In what type of area do you practice?

1. Rural area (small town or sparsely populated village)
2. Urban area (medium or large, densely populated city)
3. Suburban area (mainly residential, on the outskirts of a city or large town)

QR10.

QR11. Are you…

1. Man
2. Woman
3. Other

QR12. How old are you?

QR13. Do you smoke?

1. Yes, I smoke every day
2. Yes, I smoke occasionally
3. No, I do not smoke, but I have smoked in the past
4. No, I have never smoked

QR14. Approximately, what percentage of your patients are eligible for the complementary universal health coverage (formerly CMU-C/ACS)?

QR15. On average, how many patients do you consult per day?

QR16. On average, how many hours of consultation do you work per week?

QR17. On average, how many days per week do you consult?

If you consult for 2.5 days, please enter 2.5. If necessary, round up to the nearest half day.

QR18. Generally, do you ask your patients about…

One answer per item – Random rotation of items

a) Their alcohol consumption

b) How often they engage in physical activity

c) Their smoking status

1. Systematically and repeatedly for each patient
2. Systematically and only once per patient
3. Only for certain patients you consider at risk
4. You never ask about it

QR19. If you learn that a patient smokes, do you talk to him/her about quitting?

1. Never
2. Rarely
3. Sometimes
4. Often
5. Very often
6. Always

If rarely or never talk about quitting (QR19=1 or 2 or 3)

QR20. Why do you not talk about quitting with all your smoking patients?

Maximum 2 answers – Random rotation of items except "Other"

1. I prefer to let them bring up the subject if they wish
2. Patients may become defensive
3. It is not my role to encourage smoking cessation, especially if patients have not mentioned wanting to quit
4. I am constrained by time and must focus on the reasons for their consultation
5. I am not sufficiently informed about the different types of support and treatments to which I can refer them
6. I do not think my intervention or support will have a real impact in helping them quit smoking
7. Other: ________________

Appendix 2: FINAL QUESTIONNAIRE

This is the final questionnaire of this study. It will only take a few minutes to complete.

[If intervention group] **Your answers will help us gather feedback on the tools provided to support smoking cessation.**

[If control group] **Your answers will help us better understand the practices of general practitioners regarding smoking cessation.**

There are no right or wrong answers; we simply want to learn more about your practices.

Please note that you cannot go back to change your answers. Be sure to complete the questionnaire to the end for your responses to be recorded.

The following questions refer to your last full working day.

If you have finished today's work and it was a full day, you can use today as your reference day; otherwise, use your last full working day.

QF1. How many adult patients did you see on your last full working day?

QF2. Compared to a usual day, was this number of patients…

1. Much lower
2. Slightly lower
3. Similar
4. Slightly higher
5. Much higher

If saw at least 1 patient (QF1>0)

QF3. To your knowledge, out of these [QF1] patients, how many were current smokers (occasional or daily)?

If saw at least 1 smoking patient (QF3>0)

QF4. Out of these [QF3] smoking patients, how many did you ask on that day if they currently smoked?

If saw at least 1 smoking patient (QF3>0)

QF5. Out of these [QF3] smoking patients, with how many did you discuss smoking cessation on that day?

If did not discuss smoking cessation with all smokers (QF5<QF3)

QF6. Why did you not discuss smoking cessation with all your smoking patients on that day?
Maximum 2 answers – Random rotation of items except "Other"

1. I prefer to let them bring up the subject if they wish
2. Patients may become defensive
3. It is not my role to encourage smoking cessation, especially if patients have not mentioned wanting to quit
4. I am constrained by time and must focus on the reasons for their consultation
5. I am not sufficiently informed about the different types of support and treatments to which I can refer them
6. I do not think my intervention or support will have a real impact in helping them quit smoking
7. Other: ________________

If intervention group and discussed smoking cessation with at least one smoker (QR22=1 and QF5>0)

QF7. During this last full working day, in general, what triggered the conversations about smoking cessation?

Maximum 2 answers

1. I brought up the subject spontaneously, without the help of the questionnaires sent as part of this study
2. I gave these questionnaires to patients during the consultation to start the conversation
3. Patients brought up the subject spontaneously, without mentioning the questionnaire
4. Patients brought up the subject spontaneously because they saw the questionnaire in the waiting room, without completing it
5. Patients gave me the completed questionnaire and we discussed the subject together
6. Other: ________________

If control group and discussed smoking cessation with at least one smoker (QR22=2 and QF5>0)

QF8. During this last full working day, in general, what triggered the conversations about smoking cessation?

Random rotation of items except "Other"

1. I brought up the subject
2. Patients brought up the subject
3. Other: ________________

If smoking cessation was discussed (QF5>0)

QF9. To how many of these [QF5] smoking patients seen on your last full working day did you offer nicotine replacement therapies? (patches, gums, etc.)

If smoking cessation was discussed (QF5>0)

QF10. To how many of these [QF5] smoking patients did you offer a follow-up consultation with you on smoking?

If smoking cessation was discussed (QF5>0)

QF11. How many of these [QF5] smoking patients did you refer to another professional for smoking cessation support?

If referred at least one patient to another professional (QF11>0)

QF12. To which professional(s) did you refer these smoker(s)?

Multiple answers possible. Random rotation of items except "Other" – Items 3 and 4 always follow in this order

1. Pulmonologist
2. Addiction specialist
3. Tobacco specialist from Tabac info service
4. Other tobacco specialist
5. Dentist
6. Physiotherapist
7. Pharmacist
8. Psychiatrist
9. Psychologist
10. Nurse
11. Alternative medicine practitioner
12. Smoking cessation support association
13. Other: ________________

QF13. OVER THE LAST 2 WEEKS YOU WORKED, among the smoking patients you saw, for what proportion of them did you…

One answer per item – Random rotation of items

a) Assess their level of dependence

b) Assess their motivation to quit

c) Suggest reducing consumption for those who did not want to quit smoking

d) Remind them of the benefits of quitting smoking

1. No patients
2. A minority (less than 10%)
3. A few patients (10% to 24%)
4. Some patients (25% to 49%)
5. Most patients (50% to 74%)
6. The majority of patients (75% to 90%)
7. All patients

QF14. Please feel free to share any comments or details about how you discussed smoking cessation with your patients.

If intervention group (QR22=1)

The following questions concern the various tools and materials we sent you:

QF15. Which of these tools and materials did you receive?

Multiple answers possible except "I did not receive anything"

1. The information sheet for doctors
2. The patient questionnaires
3. The poster for the waiting room
4. The display stand
5. I did not receive anything

If received the display stand (QF15=4)

QF15b. Did you use the display stand to make the patient questionnaires available?

1. Yes
2. No

If received the information sheet (QF15=1)

QF16. Which statement best represents your experience with the information sheet?

1. I did not read the information sheet
2. I read the information sheet, but did not keep or use it afterwards
3. I read it and used it occasionally during conversations with my patients about smoking cessation
4. I read it and used it for most conversations with my patients about smoking cessation

If did not read the information sheet (QF16=1)

QF17. Why did you not read the information sheet?

Multiple answers possible – Random rotation of items except "Other"

1. I forgot to read it
2. I did not have time to read it
3. It did not seem useful to me
4. Other: ________________

If received the information sheet (QF15=1)

QF18. Please take a few seconds to read or re-read the information sheet. How do you find it in terms of…

One answer per item

a) Comprehensibility

b) Usefulness?

1. Not at all
2. Not really
3. Rather
4. Very much

If received the information sheet (QF15=1)

QF19. Do you think it contains the right level of information?

1. No, it does not contain enough information
2. No, it contains too much information
3. Yes, it contains the right level of information

If received the information sheet (QF15=1)

QF20. What other feedback or suggestions do you have about this information sheet?

If received the patient questionnaire (QF15=2)

Now, regarding the patient questionnaire…

QF21. Which statement(s) best represent(s) your experience with this questionnaire?

Multiple answers possible – Random rotation of items except "Other"

1. I did not read this questionnaire
2. I never used this questionnaire
3. I used it in conversations with my patients to ask them questions
4. I gave it to patients during the consultation
5. I asked my secretary to distribute it to patients
6. I made it available in the waiting room
7. Other: ________________

If did not read the patient questionnaire (QF21=1)

QF22. Why did you not read the questionnaire?

Multiple answers possible – Random rotation of items except "Other"

1. I did not have time
2. I forgot
3. It did not seem useful to me
4. I already discuss the subject with my patients
5. Other: ________________

If did not use the patient questionnaire (QF21=2)

QF23. Why did you not use the questionnaire?

Multiple answers possible – Random rotation of items except "Other"

1. I did not have time to make it available to patients
2. I forgot
3. I already discuss the subject with my patients
4. Questions about smoking are already systematically asked to patients upon arrival or registration at the practice
5. There is no place to put the questionnaires in the waiting room or there is no waiting room in my practice
6. I fear that my patients may become defensive
7. I do not have time to discuss smoking with my patients during the consultation
8. Other: ________________

If used the patient questionnaire (QF21=3 or 4 or 5 or 6)

QF24. In general, what proportion of your smoking patients responded to the questionnaire?

1. All
2. A majority
3. About half
4. A minority
5. None

If used the patient questionnaire (QF21=3 or 4 or 5 or 6)

QF25. What is your view on the patient questionnaire?

One answer per item – Random rotation of items

a) It is well received by my patients

b) It helps me easily identify patients who smoke

c) It helps me identify patients who want to quit smoking

d) It saves me time during consultations

1. Not at all
2. Not really
3. Rather
4. Very much

If read or used the patient questionnaire (QF21=2 or 3 or 4 or 5 or 6 or 7)

QF26. What other feedback or suggestions do you have about the patient questionnaire?

If received the poster (QF15=3)

Now, regarding the poster…

QF27. Did you display the poster in your waiting room?

1. Yes
2. No

If did not display the poster (QF27=2)

QF28. Why did you not display it?

Multiple answers possible – Random rotation of items except "Other"

1. I did not have time to display it
2. I forgot to display it
3. I have no place to display it or am not allowed to display it
4. I did not find it relevant
5. I did not know it was meant to be displayed
6. Other: ________________

If read the information sheet (QF16=2 or 3 or 4)

QF29. Would you recommend THE INFORMATION SHEET to other general practitioners? Please click on the scale to give a rating.

If read or used the patient questionnaire (QF21=3 or 4 or 5 or 6 or 7)

QF30. Would you recommend to other general practitioners to use THE PATIENT QUESTIONNAIRE? Please click on the scale to give a rating.

If received the poster (QF15=3)

QF31. Would you recommend to other general practitioners to display THE POSTER in their waiting room? Please click on the scale to give a rating.

If would not recommend one of the documents (QF29<7 or QF30<7 or QF31<7

QF32. Why would you not recommend [the advice sheet, the patient questionnaire, the poster]?

If would recommend one of the documents (QF29>6 or QF30>6 or QF31>6)

QF33. What aspects did you prefer [about the advice sheet, the questionnaire, the poster]?

If received at least one of the 3 documents (QF15=1 or 2 or 3)

QF34. If you have any other feedback or comments, please write them below.

Appendix 3: CONTENT OF THE KIT FOR GENERAL PRACTIONERS

Patient questionnaire

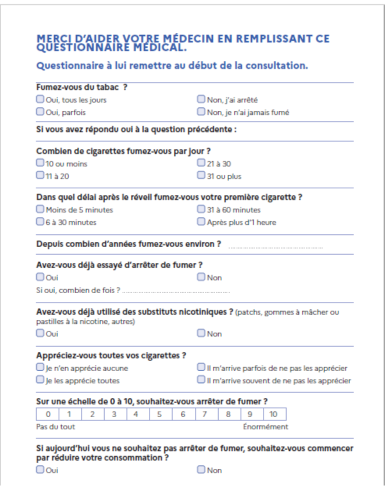

MERCI D'AIDER VOTRE MÉDECIN EN REMPLISSANT CE QUESTIONNAIRE MÉDICAL.

Questionnaire à lui remettre au début de la consultation.

Fumez-vous du tabac ?
- ☐ Oui, tous les jours
- ☐ Non, j'ai arrêté
- ☐ Oui, parfois
- ☐ Non, je n'ai jamais fumé

Si vous avez répondu oui à la question précédente :

Combien de cigarettes fumez-vous par jour ?
- ☐ 10 ou moins
- ☐ 21 à 30
- ☐ 11 à 20
- ☐ 31 ou plus

Dans quel délai après le réveil fumez-vous votre première cigarette ?
- ☐ Moins de 5 minutes
- ☐ 31 à 60 minutes
- ☐ 6 à 30 minutes
- ☐ Après plus d'1 heure

Depuis combien d'années fumez-vous environ ? ……………………

Avez-vous déjà essayé d'arrêter de fumer ?
- ☐ Oui
- ☐ Non

Si oui, combien de fois ? ……………………

Avez-vous déjà utilisé des substituts nicotiniques ? (patchs, gommes à mâcher ou pastilles à la nicotine, autres)
- ☐ Oui
- ☐ Non

Appréciez-vous toutes vos cigarettes ?
- ☐ Je n'en apprécie aucune
- ☐ Il m'arrive parfois de ne pas les apprécier
- ☐ Je les apprécie toutes
- ☐ Il m'arrive souvent de ne pas les apprécier

Sur une échelle de 0 à 10, souhaitez-vous arrêter de fumer ?

0	1	2	3	4	5	6	7	8	9	10

Pas du tout … Énormément

Si aujourd'hui vous ne souhaitez pas arrêter de fumer, souhaitez-vous commencer par réduire votre consommation ?
- ☐ Oui
- ☐ Non

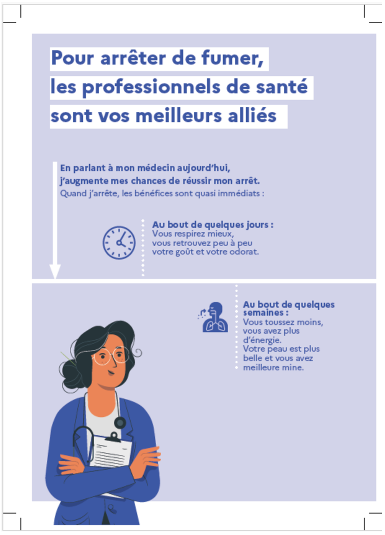

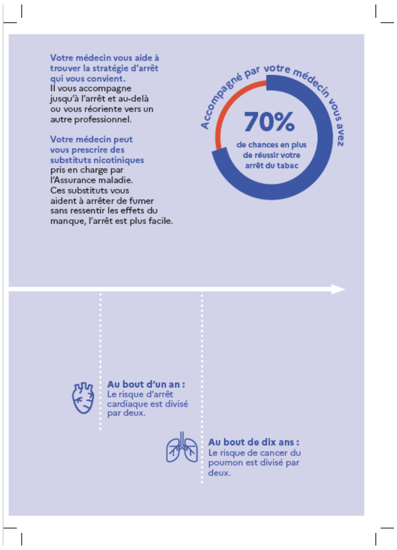

GP information sheet

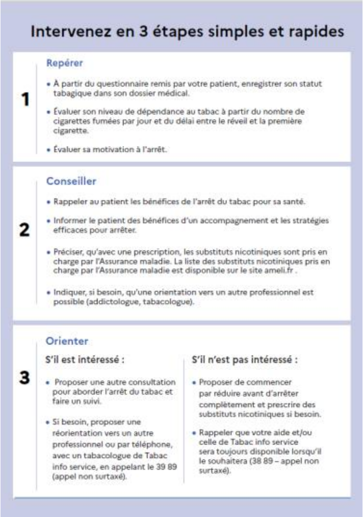

Intervenez en 3 étapes simples et rapides

1 Repérer
- À partir du questionnaire remis par votre patient, enregistrer son statut tabagique dans son dossier médical.
- Évaluer son niveau de dépendance au tabac à partir du nombre de cigarettes fumées par jour et du délai entre le réveil et la première cigarette.
- Évaluer sa motivation à l'arrêt.

2 Conseiller
- Rappeler au patient les bénéfices de l'arrêt du tabac pour sa santé.
- Informer le patient des bénéfices d'un accompagnement et les stratégies efficaces pour arrêter.
- Préciser, qu'avec une prescription, les substituts nicotiniques sont pris en charge par l'Assurance maladie. La liste des substituts nicotiniques pris en charge par l'Assurance maladie est disponible sur le site ameli.fr .
- Indiquer, si besoin, qu'une orientation vers un autre professionnel est possible (addictologue, tabacologue).

3 Orienter

S'il est intéressé :
- Proposer une autre consultation pour aborder l'arrêt du tabac et faire un suivi.
- Si besoin, proposer une réorientation vers un autre professionnel ou par téléphone, avec un tabacologue de Tabac info service, en appelant le 39 89 (appel non surtaxé).

S'il n'est pas intéressé :
- Proposer de commencer par réduire avant d'arrêter complètement et prescrire des substituts nicotiniques si besoin.
- Rappeler que votre aide et/ou celle de Tabac info service sera toujours disponible lorsqu'il le souhaitera (38 89 – appel non surtaxé).

Poster

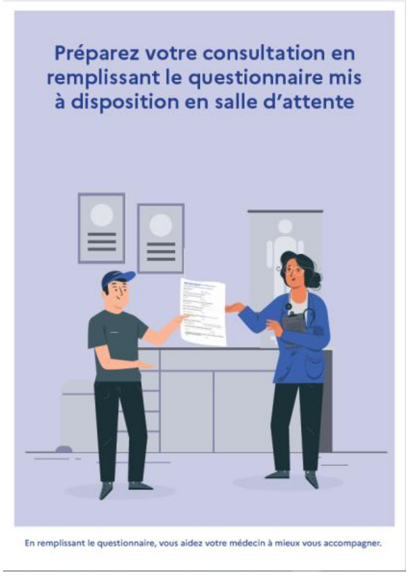

© Storyset, Freepik Company
